# Recovery and Separation of Rare Earth Elements Using Salmon Milt

**DOI:** 10.1371/journal.pone.0114858

**Published:** 2014-12-09

**Authors:** Yoshio Takahashi, Kazuhiro Kondo, Asami Miyaji, Yusuke Watanabe, Qiaohui Fan, Tetsuo Honma, Kazuya Tanaka

**Affiliations:** 1 Department of Earth and Planetary Science, The University of Tokyo, Hongo 7-3-1, Bukyo-ku, Tokyo, 113-8654, Japan; 2 Department of Earth and Planetary Systems Science, Hiroshima University, Higashi-Hiroshima, Hiroshima, 739-8526, Japan; 3 Aisin Cosmos R&D Co., LTD., Kisarazu, Chiba, 292-0818, Japan; 4 SPring-8, Japan Synchrotron Radiation Research Institute (JASRI), Sayo-cho, Hyogo, 679-5198, Japan; 5 Institute for Sustainable Sciences and Development, Hiroshima University, 1-3-1 Kagamiyama, Higashi-Hiroshima, Hiroshima, 739-8530, Japan; Reader in Inorganic Chemistry, United Kingdom

## Abstract

Recycling rare earth elements (REEs) used in advanced materials such as Nd magnets is important for the efficient use of REE resources when the supply of several REEs is limited. In this work, the feasibility of using salmon milt for REE recovery and separation was examined, along with the identification of the binding site of REEs in salmon milt. Results showed that (i) salmon milt has a sufficiently high affinity to adsorb REEs and (ii) the adsorption capacity of the milt is 1.04 mEq/g, which is comparable with that of commercial cation exchange resin. Heavier REEs have higher affinity for milt. A comparison of stability constants and adsorption patterns of REEs discussed in the literature suggests that the phosphate is responsible for the adsorption of REE in milt. The results were supported by dysprosium (Dy) and lutetium (Lu) L_III_-edge extended x-ray absorption fine structure (EXAFS) spectroscopy. The REE-P shell was identified for the second neighboring atom, which shows the importance of the phosphate site as REE binding sites. The comparison of REE adsorption pattern and EXAFS results between the milt system and other adsorbent systems (cellulose phosphate, Ln-resin, bacteria, and DNA-filter hybrid) revealed that the coordination number of phosphate is correlated with the slope of the REE pattern. The separation column loaded with milt was tested to separate REE for the practical use of salmon milt for the recovery and separation of REE. However, water did not flow through the column possibly because of the hydrophobicity of the milt. Thus, sequential adsorption–desorption approach using a batch-type method was applied for the separation of REE. As an example of the practical applications of REE separation, Nd and Fe(III) were successfully separated from a synthetic solution of Nd magnet waste by a batch-type method using salmon milt.

## Introduction

Rare earth elements (REEs) have been used in various advanced materials, including catalysts, alloys, magnets, optics, and lasers, for more than a decade [1]. In particular, the use of neodymium (Nd), sometimes with dysprosium (Dy), in high-performance permanent magnetic materials (e.g., Nd magnets) is important in terms of the amount used in the production. Although the crustal abundances of REEs [2] from 33 mg/kg (cerium) to 0.30 mg/kg (lutetium (Lu)) are higher than those of other rare metals (e.g., 10^−3^ mg/kg for palladium and much lower for platinum), the supply of several REEs is sometimes limited partly for “geopolitical” reasons [1]. Thus, recycling REEs is important because global in-use stocks are almost four times of the 2007 annual production rate for various REEs [3].

REE extraction from aqueous solution is an important step to recover REE from their wastes if the REEs can be properly dissolved into a solution [4]. Solvent extraction has been developed as an effective method since the 1930s among various techniques [5]. It is currently one of the main techniques used to recover REEs from aqueous solutions and to perform mutual separation of REEs. Although all REEs coexist in natural mineral ores, each REE is used separately in such applications. Thus, mutual separation of each REE is crucial to various industrial applications. However, the solvent extraction method has some shortcomings, including (i) the use of harmful reagents such as strong acids and organic solvents and (ii) the high cost of chemicals such as extractants [6]–[8].

DNA attached to cellulose (DNA-filter hybrid) has been successfully employed for the recovery and separation of REEs through column method, where the binding site of REEs is the phosphate within the DNA [9]. The high affinity of REEs for phosphate has also been found in adsorption studies of REEs on bacteria [10]–[12]. In the present research, we examined the feasibility of REE recovery from aqueous solution by salmon milt, given that salmon milt is one of the original materials of DNA that is commercially available as reagent that is widely used in scientific research. Salmon milt can function as an adsorbent to REEs because the DNA exposed out of the milt can serve as the binding site of REEs. Moreover, more than 10,000 tons/year of milt from salmon, trout, and others have been discarded as industrial wastes from fishery industries in Hokkaido, Japan [13]. Thus, the cost of using milt is low, which enables us to use milt as a cost-effective material for the recovery of REEs or even of other metal ions. In addition, milt is an environmentally friendly biomaterial that has no hazardous effects on the environment after use.

The presence of anionic functional groups in milt can contribute to its binding to metal ions. For DNA, researchers have conducted various studies on the removal of inorganic and organic substances using DNA-based materials [14]–[17]. Our previous studies on the interaction of REEs with bacteria and DNA suggest high affinities of REEs to phosphate sites in bacterial cell surfaces or DNA [9]–[12]. Based on these findings, we considered that milt also has high affinity to REEs through the phosphate sites. However, no efforts have been devoted in using milt for the separation of REEs, although its cost is much lower than that of DNA.

We applied salmon milt to remove REEs from an aqueous solution in this study. The column separation method could not be used because of the hydrophobic nature of milt, which prevented water from flowing through the column loaded with milt. Thus, we used an adsorption–desorption process by means of a batch-type method for the separation. Adsorption capacity and pH dependence were examined to characterize milt as an adsorbent of REE. The binding site of REEs in DNA was also examined through extended x-ray absorption fine structure (EXAFS) spectroscopy [18]. To characterize salmon milt as adsorbent, we compared its binding site with those of cellulose phosphate (CP), carboxymethyl cellulose (CMC), Ln-resin (ion-exchange resin having organophosphate ligand), bacteria surface, and DNA-filter hybrid that were studied in our previous studies. Finally, batch method using milt was applied to separate Nd, Dy, and trivalent iron (Fe(III)), the main metals employed in Nd magnet (Nd_2_Fe_14_B). We did not study the separation of REEs from other transition metals because we focused on Nd, Dy, and Fe by taking account of its strong application in magnet industries.

Although many studies apply biomaterials to recover and separate metal ions, we have developed this idea because REEs favor phosphate sites of bacterial cell surfaces and DNA. DNA is industrially produced from salmon milt, which is sold as reagents and industrial materials. For example, salmon milt-based DNA has been reported to be used for a template for mass production of silver nanoparticles [19]. Thus, various applications of salmon milt have been developed in various fields, including this study.

## Experimental

### Materials and preparation of milt powder

Salmon milt from salmon originally caught in Okhotsk Sea, Hokkaido, Japan was purchased from Izumi Co., Ltd. (Hiroshima, Japan) and used in this study. The salmon milt from Hokkaido has been used for wide applications in various fields [19]–[21]. In our preliminary study, we also used salmon milt from Miyagi Prefecture, which basically showed similar results as reported in this manuscript. Salmon milt washed with Milli-Q water was fragmented into small pieces (approximately. 2 cm×2 cm) and freeze-dried. Afterward, the milt was powdered using an agar mortar. The obtained milt powder was used in further experiments. The powdered milt can be preserved without degradation for more than half a year even at room temperature. The presence of phosphate functional groups in milt was confirmed through BIOMOL Green method [22]. The particle size of the milt was 30 µm to 80 µm based on observation from scanning electron microscopy (SEM; JEOL).

We used a mixed standard solution that contains all the REEs (except promethium (Pm)) and standard solutions that contain Nd (SpexCertiPrep, New Jersey, USA) after dilution to appropriate concentrations. Ln-resin (2.0 mEq/g; Eichrom) [23] and cellulose phosphate (CP; 1.08 mEq/g; Sigma, USA) were used as reference materials that can adsorb REEs through phosphate as the binding site within the cellulose structure [12]. We also used carboxymethyl cellulose (CMC; 0.60 mEq/g; Sigma, USA) as a material for binding to REEs through carboxylate for comparison [12]. These reference materials were considered the analogs of biological macromolecules with phosphate and carboxylate as binding sites of metal ions. All other reagent-grade chemicals were used as received.

### Adsorption experiment

A batch experiment was conducted to study REE adsorption onto the salmon milt. To determine the pH dependence of the adsorption behavior, 10 mL of the solution containing all REEs (1 mg/L each REE) was mixed with 20 mg of powdered milt. The ionic strength was adjusted to 0.010 M using NaCl. The pH was adjusted to a specific value by adding a small amount of HCl or NaOH solution. The reaction time was 3 h, which was long enough to attain equilibrium, supported by our preliminary experiments for the time dependence examined before the batch experiment. The adsorption capacity of milt was also determined in a batch experiment, in which the solution at various concentrations of Nd was mixed with 0.20 g of milt. The dependence of the adsorption amount of Nd on the equilibrium concentration in water was examined to determine the adsorption capacity of Nd on milt.

### Extended x-ray absorption fine structure (EXAFS) spectroscopy

EXAFS spectra were recorded for Dy and Lu adsorbed onto the milt to confirm the binding site of REEs on the milt. A similar experiment has been reported by Takahashi et al. (2010, 2012) [9], [12]. The sample for the milt was prepared at constant concentration of each REE (Dy and Lu) as 1×10^−7^ mol/mg milt. The local structures of REEs adsorbed onto Ln-resin, CP, and CMC were also studied as reference materials. As an example of REE, Dy L_III_-edge EXAFS spectra were recorded in beamline BL14B2 or BL01B1 at SPring-8 (Hyogo, Japan). The two beamlines have similar x-ray sources (i.e., bending magnet) and layouts of optics. The x-rays were monochromatized with a pair of Si(111) crystals. The beam size of the x-ray at the sample position was approximately 1×4 mm^2^, and its incident intensity (I_0_) was monitored with an ionization chamber. The sample was placed at 45° from the incident beam, and the fluorescent x-rays were measured with a 19-element Ge solid-state detector (SSD) to obtain the spectra of Dy on the milt and reference materials.

**Figure 1:**
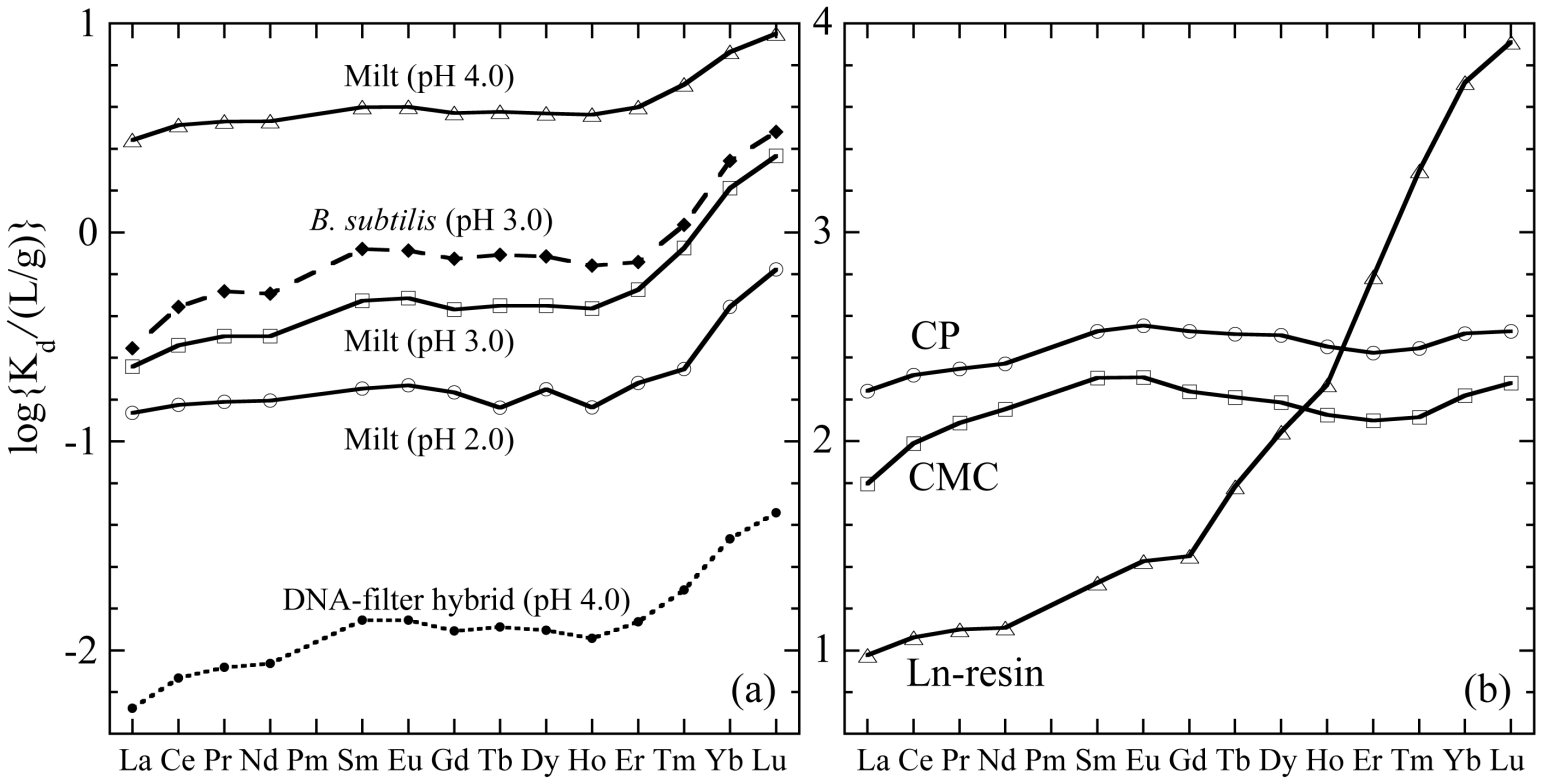
REE patterns of distribution coefficients (*K_d_*, L/g) for REE adsorption. (a) salmon milt (pH 4, 3, and 2), bacteria (*B. subtilis*) [10], and DNA-filter paper hybrid [9]; (b) CP, CMC, and Ln-resin [9], [10].

The spectra at Lu L_III_-edge and Nd L_III_-edge were also measured at BL12C at the Photon Factory (Tsukuba, Japan). However, the signal-to-noise (S/N) ratio of the spectra obtained at Nd L_III_-edge was low, possibly because of the lower adsorption amount of Nd compared with Dy and Lu (Fig. 1). Thus, the structural data of Nd adsorbed on the milt cannot be obtained by EXAFS analysis. Beamline BL12C is also a hard x-ray XAFS station. The measurement was conducted in fluorescence mode using a 19-element Ge-SSD, as described earlier. A total of one to three scans were added to improve the S/N ratio, and all of the spectra were normalized to unit steps in the absorption coefficient. No radiation damage was found during data acquisition because multiple scans provided identical spectra.

EXAFS data analysis was performed using REX 2000 codes (Rigaku Co., Ltd.). Phase shifts and amplitude functions for data simulation were calculated using FEFF7.0 [24] for each element based on the structures of LnPO_4_ (Ln  =  Dy or Lu) [25]. The experimental EXAFS function χ(k) was obtained after subtracting the embedded-atom absorption background from the Lα fluorescence signal of each REE to the intensity of the incident beam (I_0_). EXAFS spectra were Fourier transformed (FT) after multiplying the Hanning function to minimize truncation effects at both ends of the k region subject to FT. FT-EXAFS spectrum, that is, the radial structural function (RSF), was back-transformed to k space for neighboring shells of interest in the RSF for spectral simulation using the parameters extracted with FEFF7.0.

### Recovery of Nd, Dy, and Fe by leaching from milt

In this study, we used a mixture solution of Fe^3+^ (11.0 mg/kg), Nd (5.0 mg/kg), and Dy (2.0 mg/kg), which simulates a solution obtained from the decomposition of Nd magnet (ionic strength: 0.010 M by NaCl). The initial pH was adjusted to 4 using a small amount of NaOH or HCl solution. Up to 10 mL of the solution was mixed with 0.020 g of milt powder in a polyethylene centrifuge tube and was shaken for 1 h, where more than 99% of Fe, Nd, and Dy was adsorbed on the milt. After 1 h, the milt powder and the solution were separated by centrifugation (2000 rpm, 10 min). The recovered milt powder was mixed with 10 mL of HCl solution (pH 2). After the subsequent separation of milt and water, the concentrations of Fe, Nd, and Dy that leached into the solution were measured through ICP-AES (Fe) and ICP-MS (Nd and Dy). The leached fractions of Nd, Dy, and Fe relative to the initial amount adsorbed on the milt were lower than 80%, so the leaching procedure of adding 10 mL of HCl solution (pH 2) into the centrifuge tube with the remaining milt powder was repeated. The total amounts of Nd, Dy, and Fe recovered into the solution relative to their initial amounts were determined through ICP-AES and ICP-MS measurements.

## Results and Discussion

### Adsorption of REE on the milt

The adsorption of REEs onto milt was examined at different pH values (Fig. 1). The REE pattern of *K_d_* (solid–water distribution coefficient, L/g) was larger at heavy REE part (HREE) such as Tm, Yb, and Lu. This pattern is similar to those observed for REE adsorption onto bacterial cell surfaces [10] and DNA (DNA–filter hybrid) [9], as shown in Fig. 1a (for comparison, the REE patterns of *K_d_* for Ln-resin, CP, and CMC are shown in Fig. 1b). The *K_d_* value of DNA is much lower than those of other systems because of the lower cation exchange capacity of the material whose weight is dominated by the filter which is not responsible for the adsorption of REE. However, the dependence of the *K_d_* value on the REE atomic number (REE pattern of *K_d_*) is important, given that this dependence relies on the relative variation in the stabilities of REE species adsorbed on the adsorbents. For example, the REE pattern of *K_d_* can change according to the surface complexes of REE, such as phosphate and carboxylate on solid surfaces or adsorbed as hydrated species (outer-sphere complexation) [12], [26], [27]. The similarity of the REE pattern between milt and bacteria/DNA implies that phosphate, which is the main binding site of REE to bacteria/DNA, can also be the binding site in the milt. This scenario will be confirmed by the EXAFS analysis described in Section 3.3.

The characteristics of the REE pattern for adsorption onto the milt are as follows: (i) the adsorption in the light REE (LREE) region from La to Nd is weaker than that in other REE regions and (ii) the middle REE (MREE) components, such as Sm and Eu, are more strongly adsorbed compared with other REEs, except for the HREE components. The mutual difference among the REEs suggests that the three subgroups [i.e., LREE (La, Ce^3+^, and Pr), MREE (Sm and Eu), and HREE (Tm, Yb, and Lu)] can be roughly separated based on the differences in the REE affinity to the milt. In particular, adsorption onto the milt can specifically isolate Yb and Lu because of the larger differences in their affinities to the milt compared with other REEs.

REE adsorption became weaker as the pH decreased (Fig. 1), which is frequently observed in the adsorption behavior of cations on deprotonated binding sites [28]. Thus, the adsorption–desorption behavior at different pH levels can be used to separate REEs as usually conducted in the ion-exchange method for separating different ions. The difference in *K_d_* from lighter to heavier REEs was larger at a lower pH value (i.e., pH 2 and 3) compared with that at pH 4. The ratio of K_d_(Lu)/K_d_(La) was 3.2 at pH 4, which was smaller than those at pH 3 (10) and pH 2 (4.8). This result suggests that desorption at lower pH regions can enhance the effectiveness of the mutual separation of REEs after initial adsorption at higher pH.

### Adsorption capacity

**Figure 2:**
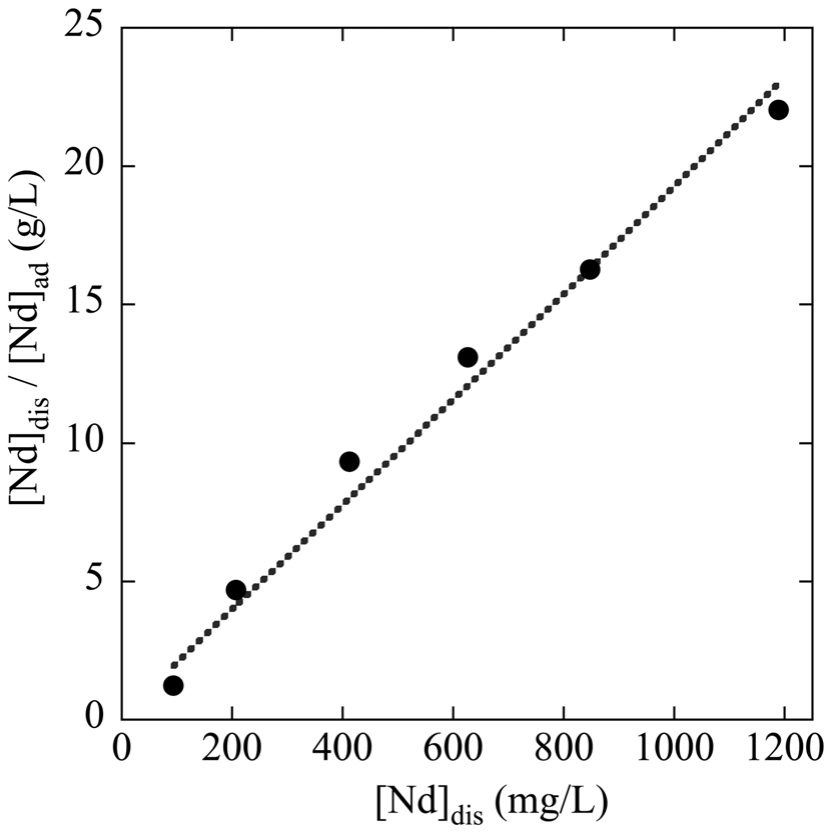
Relationship between [Nd]_dis_ and [Nd]_dis_/[Nd]_ad_ (Langmuir function). [Nd]_dis_ and [Nd]_ad_ denote the equilibrium concentrations of Nd in the aqueous and solid (salmon milt powder) phases, respectively, obtained at various initial Nd concentrations. A linear line obtained by least-square fitting is also shown (r^2^ = 0.99992) to estimate the maximum adsorption of Nd onto the salmon milt powder.

The adsorption isotherm was examined by the Nd adsorption onto the milt at different initial concentrations at pH 3.5 and 25°C (Fig. 2). The relationship between [Nd]_dis_ (Nd concentration in the aqueous phase) and [Nd]_dis_/[Nd]_ad_ (where [Nd]_ad_ is the Nd concentration in the solid phase) is shown. The Langmuir isotherm [28] can be written as follows:
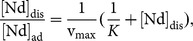
where *K* and *v*_max_ are the equilibrium constant and amount of maximum adsorption, respectively. The successful fitting of the relationship between [Nd]_dis_ and [Nd]_dis_/[Nd]_ad_ with a line by least-square analysis (*r* ^2^ = 0.986) shows that the reaction obeys the Langmuir-type adsorption. The fitting shows that the maximum adsorption amount was 50.1 Nd mg/g or 1.04 mEq/g. This value is comparable with that of normal strongly acidic cation exchange resin (typically ca. 1 mEq/g). Considering that salmon milt is an industrial waste [13], the use of milt can be a cost-effective method to recover REEs and other cations. Moreover, milt is an environmentally friendly material and no resources are required to produce milt as a separation material.

### Binding site of REE examined by EXAFS

**Figure 3:**
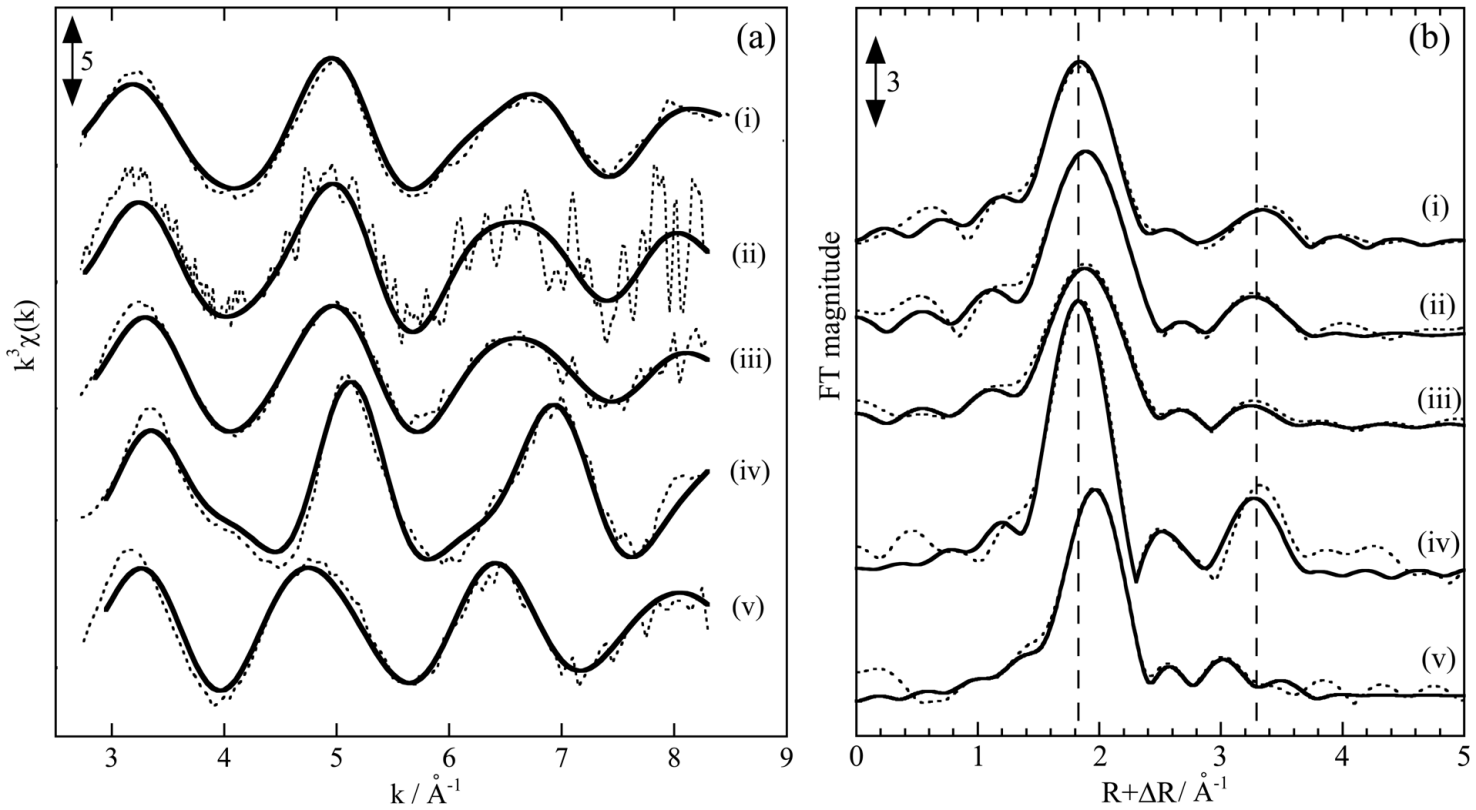
EXAFS spectra at Dy L_III_-edge adsorbed on various adsorbents. Spectra in (a) k- and (b) R- spaces are shown for Dy adsorbed on (i) milt powder (pH 3), (ii) DNA-filter hybrid (pH 3), (iii) CP (pH 4), (iv) Ln-resin (pH 4), and (v) CMC (pH 4). The dotted and closed curves show the experimental and simulated results, respectively.

We have measured the L_III_-edge EXAFS of Nd, Dy, and Lu adsorbed on the salmon milt to represent light, middle, and heavy REEs, respectively. However, Nd L_III_-edge EXAFS data were not provided here because of the poor spectra quality caused by the lower Nd distribution to the milt compared with Dy and Lu. Fig. 3 shows the EXAFS spectra obtained at Dy L_III-_edge for k- and R-spaces. The spectra of Dy adsorbed onto the milt are provided along with those of Dy on the DNA-filter hybrid and other reference materials. The reference materials include (i) Ln-resin and CP with phosphate as the binding site and (ii) CMC with carboxylate. Takahashi et al. [9], [12] reported that the binding site in Ln-resin and CP for REEs is phosphate reflected by the peak found at R+ΔR = 3.3 (Å) in the R-space spectra of Dy (Fig. 3b). The fitting of the spectra by the FEFF parameters generated shows that the interatomic distances between Dy and P were 3.76 Å for Dy on both CP and Ln-resin. Although the phosphate is the binding site for both adsorbents, the coordination number of phosphate groups (CN) bound to Dy on CP was 1.1, which is smaller than that for Dy on Ln-resin (CN = 7.2). The difference in CN can affect the average distance between Dy and oxygen in the adsorbents. The average Dy-O distances were 2.33 Å and 2.25 Å for Dy on CP and Ln-rein, respectively. This difference can be caused by two factors: (i) the Dy-O distance for Dy bound to phosphate is smaller than that for the water molecule bound to Dy, and (ii) the ratio of water molecules remaining in the first coordination sphere for CP was larger than that for Ln-resin. Thus, the peak of Dy-O in the R-space shifts to a larger R for CP compared with that in the Ln-resin system. The EXAFS spectrum of Dy on DNA-filter paper was similar to that on CP, which shows a similar coordination environment between the two systems.

The EXAFS spectra of adsorbed Dy for the milt (pH 3) were almost identical to those of Dy on CP (pH 3) both in k- and R-spaces. The Dy-O and Dy-P peaks found in the RSF for Dy on the milt (Fig. 3b) were at the same peak positions for Dy adsorbed on CP. This result shows that the phosphate group in the milt is the REE binding site. The EXAFS parameters obtained for the samples (Table 1) confirm that the coordination environment of Dy adsorbed onto the milt is similar to that on CP.

**Table 1:** EXAFS parameters for Dy and Lu species adsorbed on salmon milt and reference materials (CN, coordination number; R, interatomic distance; ΔE0, threshold E0 shift; σ, Debye-Waller factor; least squares precisions are given to each value).

Sample	k range (Å^−1^)	Shell	CN^a^	R (Å)^a^	ΔE_0_ (eV)	σ^2^ (x10^3^ Å^2^)^a^^,^ b	Residual (%)
Dy on CP	2.85–8.85	Dy-O	10.2±1.1	2.329±0.020	4.8±2.1	12.1±0.2	1.6
		Dy-P	1.1±0.1	3.759±0.111		12.1±0.9	
Dy on CMC	2.95–10.75	Dy-O	10.9±1.0	2.383±0.020	3.2±2.5	12.1±0.2	0.9
		Dy-C	2.7±0.1	3.401±0.19		5.3±0.9	
Dy on Ln resin	2.80–10.35	Dy-O	9.6±2.1	2.249±0.016	3.9±2.0	7.6±0.8	1.4
		Dy-P	7.2±0.1	3.762±0.104		13.7*	
Dy on DNA-filter hybrid, pH 3	2.66–9.38	Dy-O	11.9±2.8	2.343±0.020	2.7±2.1	12.1	0.8
		Dy-P	1.2±0.2	3.742±0.027		12.1*	
Dy on salmon milt, pH 3	2.75–9.45	Dy-O	10.5±1.1	2.308±0.019	2.2±2.0	11.0	1.0
		Dy-P	2.1±0.1	3.795±0.027		6.7±0.9	
Lu on CP	2.75–10.2	Lu-O	9.8±2.3	2.284±0.019	6.8±2.1	12.1±0.8	3.3
		Lu-P	2.3±0.1	3.812±0.050		12.1*	
Lu on CMC	2.80–10.35	Lu-O	10.1±2.3	2.298±0.019	6.3±2.1	10.8±0.8	1.3
		Lu-C	3.6±0.1	3.328±0.050		4.9*	
Lu on Ln resin	2.80–10.35	Lu-O	8.4±1.7	2.184±0.014	6.4±2.1	5.8±0.8	1.4
		Lu-P	5.3±0.3	3.734±0.025		9.6±0.8	
Lu on DNA-filter hybrid, pH 3	2.80–10.05	Lu-O	10.5±2.1	2.270±0.017	6.0±2.0	12.3±0.6	1.1
		Lu-P	1.9±0.1	3.786±0.195		10.0±0.6	
Lu on salmon milt, pH 3	2.90–10.40	Lu-O	8.2±1.7	2.203±0.015	6.7±2.1	6.7±0.8	1.1
		Lu-P	3.5±0.1	3.773±0.112		4.1	

a Accuracy in the fitted parameters were estimated to be generally ±0.02 Å for R, ±20% for CN, and 20% for σ. [2], [31].

b The numbers with * were fixed during the fitting of EXAFS spectra.

In addition, the EXAFS spectrum of Dy on the milt was similar to that on the DNA-filter hybrid. This result suggests that the binding site in the milt was similar to that bound to DNA. Milt contains a large amount of DNA. Although DNA is encapsulated in each cell or mainly a sperm in the milt, DNA with phosphate may be exposed to bulk water in milt suspension, which can be accessed by REE in the solution.

**Figure 4:**
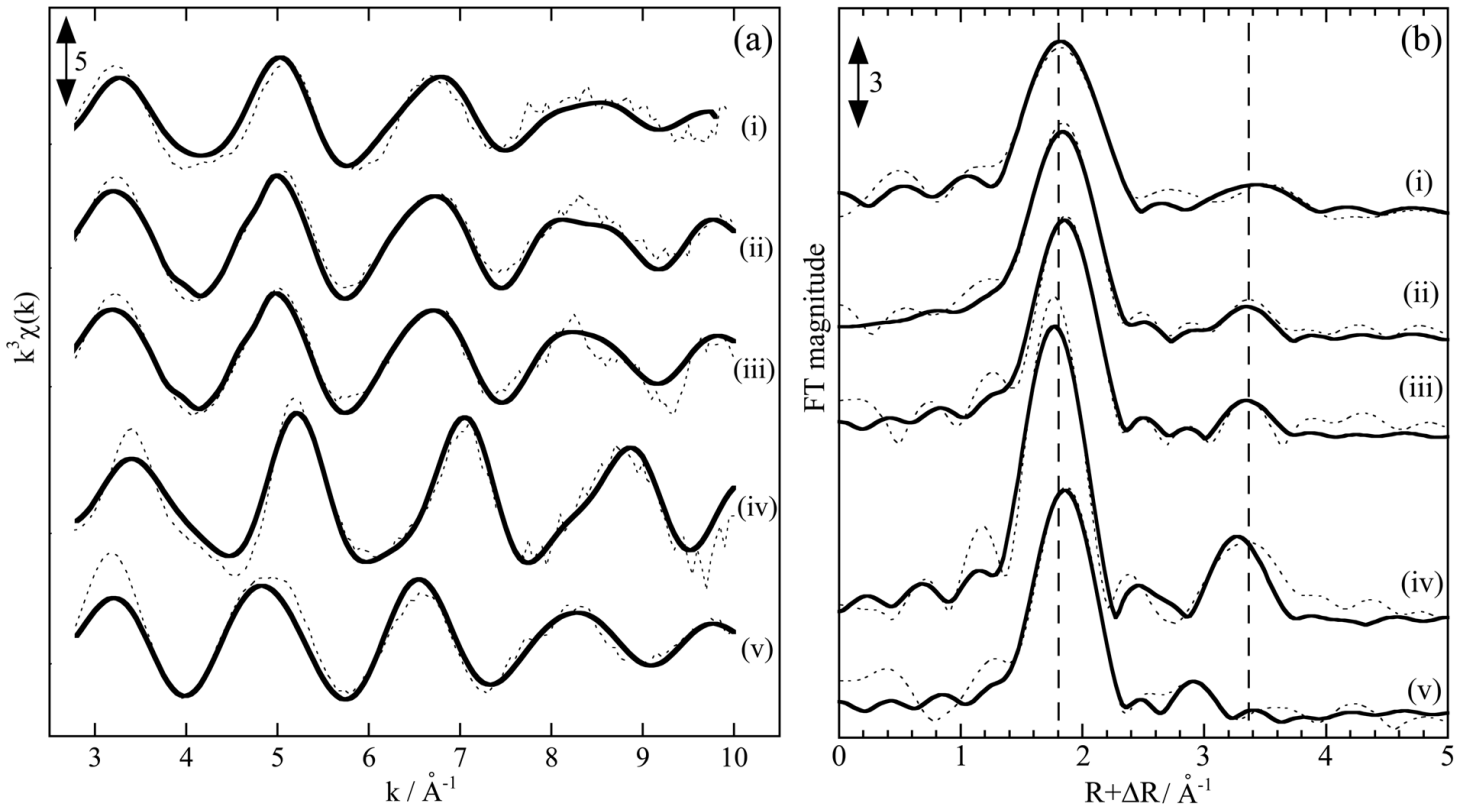
EXAFS spectra at Lu L_III_-edges on various adsorbents. Spectra in (a) k- and (b) R- spaces are shown for Lu adsorbed on (i) milt powder (pH 3), (ii) DNA-filter hybrid (pH 3), (iii) CP (pH 4), (iv) Ln-resin (pH 4), and (v) CMC (pH 4). The dotted and closed curves show the experimental and simulated results, respectively.

Similar EXAFS data were obtained for Lu (Fig. 4; Table 1), which shows that the spectra of Lu adsorbed on the milt were similar to those adsorbed onto CP. These results reveal that the phosphate site is mainly responsible for the adsorption of all REEs onto the milt and DNA-filter hybrid. Variations in the REE pattern of the *K_d_* value can be discussed based on the EXAFS results. For any adsorbents, the CN value provided by EXAFS is consistent with the affinities of Dy and Lu for each adsorbent. The larger CN value of Lu-P than that of Dy-P in CP, CMC, DNA-filter hybrid, and salmon milt (Table 1) supports the larger *K_d_* value of Lu than that of Dy in each system.

The MREE peak and increase in HREE found in milt and the DNA-filter hybrid (Fig. 1) were also found in the REE pattern for CP [9]. Thus, the EXAFS data that indicate the similarity among milt, DNA-filter hybrid, and CP are consistent with the REE distribution patterns, which can also reflect the properties of the REE binding sites. However, when we compared their REE patterns more precisely, the REE patterns for the milt and DNA-filter hybrid showed larger enrichment in HREE than those in CP. Previous studies show that the slope of the REE pattern can reflect the CN of the ligand binding to REE [29], [30]. The CN values of the phosphate (CN for the REE-P shell) for Dy or Lu provided in the milt system were larger than those for the respective REEs in the CP system but smaller than those in the Ln-resin system. The slope of the REE pattern for the milt system (K_d_(Lu)/K_d_(La) = 3.2–10) is larger than that in the CP system (K_d_(Lu)/K_d_(La) = 1.9), but smaller than that in the Ln-resin system (K_d_(Lu)/Kd(La) = 860), as shown in Fig. 1b. This result shows that the REE pattern is steeper for the species with larger CN values. Thus, the implication obtained by the REE pattern of the *K_d_* value for the milt is consistent with the binding-site information provided by EXAFS. The similarity between the milt and DNA-filter hybrid found in these results also confirms that DNA or a similar material is the REE binding site in the milt.

### Recovery of REE by the milt

The recovery of REEs using a column loaded with milt was initially tested, given that the column method is an effective separation method with multiple adsorption–desorption processes during the flow of the REE solution through the column. However, we found that water did not flow out through the column loaded with the milt possibly because of the hydrophobicity of the material. We performed the following processes to decrease this effect: (i) diatom earth was mixed with milt (1∶1 by weight), and (ii) ethanol was added into the solution (1∶9 ethanol∶water ratio by volume). However, these modifications did not improve the flow rate of water through the column. Thus, we concluded that the column method with milt cannot be used as a method to recover REE.

**Figure 5:**
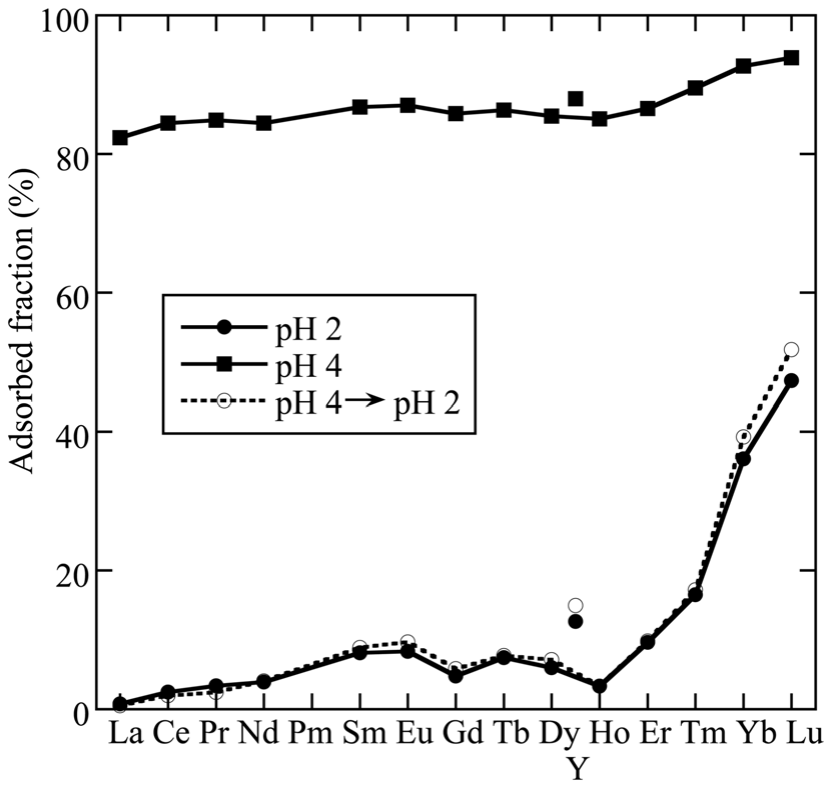
REE patterns of adsorbed fraction of REE onto the milt powder at pH 2 and pH 4. The adsorbed fraction at pH 2 after adsorption at pH 4 and its acidification to pH 2 are also shown to assess the reversibility of the adsorption.

As an alternative method, a batch process that consists of adsorption at higher pH values and subsequent desorption at lower pH values was conducted by employing milt as the adsorbent. First, the batch method was used for the solution that contained all REE. The milt was added into the REE solution that contained all REEs (i.e., Y and lanthanides except Pm, for a total of 15 elements) at pH 4 and 25°C (i.e., each REE: 0.10 mg/L; milt: 2.0 g/L). The ionic strength was adjusted to 0.010 M using NaCl, and the reaction time was 1 h. More than 80% of all REEs were adsorbed on the milt at pH 4 (Fig. 5). In particular, heavier REEs such as Tm, Yb, and Lu were strongly adsorbed onto the milt. After acidifying to pH 2, the adsorbed fraction of REE remaining in the milt was lower than 20%, except for Yb and Lu (Fig. 5). The results show that (most REE can be recovered and redissolved in water through the batch method with the milt at pH values ranging from 2 to 4, which are not very acidic in terms of experiment safety. The adsorbed fraction after acidification from pH 4 to pH 2 was similar to that in the system initially adjusted to pH 2. This result shows that the reaction is reversible and that we can recover almost all REEs through it.

**Figure 6:**
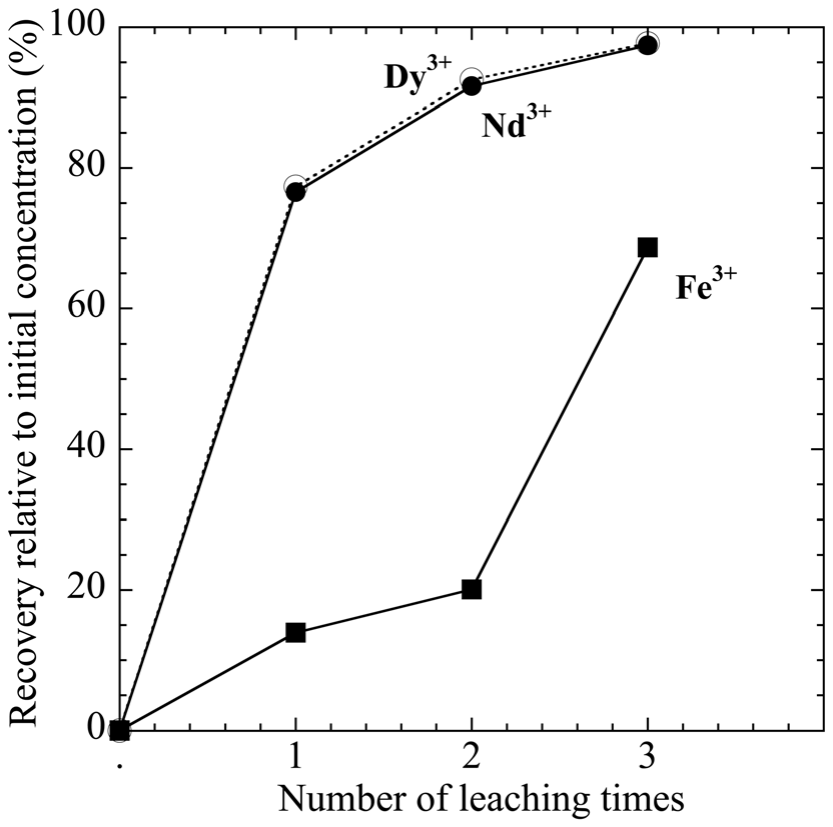
Total recovery of Nd, Dy, and Fe(III) by repetitive leaching into HCl solution at pH 2 from the milt powder after the initial adsorption of these ions on the milt at pH 4 from their solution assuming the decomposed solution of Nd magnet.

For a more practical application, we compared the recoveries of Fe, Nd, and Dy leached using 0.010 M HCl solution from the milt adsorbing the three ions (Fig. 6), where we considered the application of the method to recycle REE wastes from Nd magnets. Fig. 6 shows that the total recoveries of Nd, Dy, and Fe(III) after adsorption on the milt by multiple leaching into the HCl solution (pH 2) were plotted. The total recovered fraction including all the steps is plotted in the vertical axis in Fig. 6. However, the leaching behavior of Nd and Dy was almost identical, which shows that the mutual separation of REEs cannot be possible through the batch method, except for Yb and Lu, which behave very differently from the other REEs (Fig. 5). The column method is usually employed for precise separation of different metal ions by solid adsorbent, which cannot be applied to the method that uses milt. Thus, we concluded that milt cannot be effective for the mutual separation of REEs.

However, Fig. 6 shows that practical application for Fe and Nd (or Dy) separation is clearly possible through the batch method. Using the two treatments of the synthetic solution of Fe, Nd, and Dy, we found that more than 90% of Nd (and Dy) can be recovered, whereas only 20% of Fe was leached from the milt. Thus, milt can be used to recover REEs and even separate them from other ions such as Fe. This characteristic can be suitable for Nd and Dy recovery from the waste solution of Nd magnets that mainly contains Nd, Dy, and Fe.

## Conclusions

This study shows that freeze-dried salmon milt can be used as adsorbent for REEs and possibly other cations. The EXAFS results show that the phosphate site in the milt is responsible for REE adsorption onto the milt. The adsorption capacity of REE or cation was 1.0 mEq/g, which is comparable with that of commercial cation exchange resin. Although milt cannot be used for the column method because water does not flow through the milt-loaded column, milt powder can still be used for batch-type recovery and separation. For example, assuming its application to Nd magnet, separation of Fe and REE (Nd or Dy) is possible through adsorption on milt at pH 4 and subsequent REE desorption from the milt at pH 2. Considering that milt, an industrial waste from fishery industries, is a low-cost material, its ion-exchange capacity has a substantial potential for use in different fields such as the recovery of cations (e.g., REE) and wastewater treatment.
